# Characteristics of sarcopenia after distal gastrectomy in elderly patients

**DOI:** 10.1371/journal.pone.0222412

**Published:** 2019-09-11

**Authors:** Sadamu Takahashi, Shota Shimizu, Satoshi Nagai, Hiroshi Watanabe, Yuuko Nishitani, Yasuro Kurisu

**Affiliations:** Department of Surgery, National Hospital Organization Hamada Medical Center, Asai-tyou, Hamada, Japan; Ehime University Graduate School of Medicine, JAPAN

## Abstract

Presence of preoperative sarcopenia is a risk factor for postoperative complications. However, there are few reports on the presence of sarcopenia and its characteristics following gastrectomy. Sarcopenia is closely related to quality of life in elderly people. To date, the main purpose of follow-up after gastrectomy is surveillance for early detection of recurrence and secondary cancer. However, henceforth, quality of life in elderly gastric cancer patients after gastrectomy must also be evaluated. The present study aimed to investigate sarcopenia during a 1-year postoperative course in elderly gastric cancer patients and examine their characteristics. The subjects were 50 patients aged ≥70 years who underwent laparoscopy-assisted distal gastrectomy for gastric cancer and who experienced no recurrence 1 year postoperatively. Height, weight, serum albumin levels, food intake amount, grip strength, gait speed, visceral fat area, and appendicular skeletal muscle mass index were measured preoperatively and 6 months and 1 year postoperatively. Sarcopenia, obesity, and visceral obesity were diagnosed. Compared with preoperatively, indicators other than height decreased 6 months postoperatively. Compared with 6 months postoperatively, body weight, amount of food intake, and visceral fat area increased by 1 year postoperatively, unlike appendicular skeletal muscle mass index. The frequency of sarcopenia increased 6 months postoperatively compared with preoperatively; this frequency remained almost unchanged 1 year postoperatively compared with 6 months postoperatively. Further, the frequency of visceral obesity increased 1 year postoperatively compared with 6 months postoperatively. Weight increased after > 6 months postoperatively; however, most of the weight increase was in terms of fat and not muscle. We emphasize the importance of considering postoperative sarcopenia and visceral obesity. In particular, sarcopenia and visceral obesity should be carefully monitored after increases in body mass index and food consumption.

## Introduction

In recent years, sarcopenia has been defined as the condition in which muscle mass, muscle strength, and physical ability decline [1, 2]. Sarcopenia is closely related to quality of life in elderly people. Following the onset of sarcopenia, fracture, cardiovascular morbidity, and mortality rates reportedly increase [3–5]. In the field of surgery, the relevance of sarcopenia during the perioperative period has been evaluated. Studies have reported sarcopenia as a risk factor for operative complications and poor prognosis [6–8]. In cases of gastrectomy performed for gastric cancer, the presence of preoperative sarcopenia has been reported as a risk factor for postoperative complications [9, 10]. However, only few studies have reported the presence of sarcopenia and its long-term characteristics following gastrectomy for gastric cancer. When gastrectomy is performed in gastric cancer patients, food intake and body weight may decrease in the short term. After the first 6 months postoperatively, weight loss stops and body weight begins to increase and gradually stabilizes after 1 year postoperatively [11]. During this postoperative course, the degree/extent of changes in muscle mass, fat mass, and physical ability remain unknown.

The average life expectancy is increasing, and the number of older gastric cancer patients is increasing. Approximately half of the cases of patients who undergo gastric cancer surgery are early cancer, and the 5-year survival rate after early gastric cancer surgery is > 95% in Japan [12]. Therefore, the number of elderly people who live for a long term after gastric cancer surgery is increasing. To date, the main purpose of follow-up after gastrectomy for gastric cancer was surveillance for early detection of recurrence and secondary cancer. However, we further also need to pay attention to quality of life in elderly gastric cancer patients after gastrectomy. Therefore, the present study aimed to investigate sarcopenia during a 1-year postoperative course in elderly gastric cancer patients and examine their characteristics.

## Materials and methods

### Subjects

The subjects were 50 patients aged ≥70 years who underwent laparoscopy-assisted distal gastrectomy for the radical treatment of gastric cancer between November 2009 and October 2015 at National Hospital Organization Hamada Medical Center. Surgery was performed in accordance with the Japanese gastric cancer treatment guidelines 20104 (ver.4) [13]. Lymphadenectomy was performed for D1 or D2. The pathological stage was I for all the patients. Billroth I (an operation in which the pylorus is removed and the proximal stomach is directly anastomosed to the duodenum) was performed for reconstruction, and an automatic anastomotic device was used to create anastomoses in all the patients. The present study included only community-dwelling patients who could orally ingest and perform activities of daily living at 1 year postoperatively. Patients who underwent postoperative adjuvant chemotherapy or in whom recurrence or edema was noted were excluded. Height, weight, amount of dietary intake, grip strength, gait speed, body mass index (BMI), and corrected appendicular skeletal muscle mass index were measured by a bioelectrical impedance analyzer, and visceral fat area was measured using computed tomography images. Measurements were performed preoperatively, 6 months postoperatively, and 1 year postoperatively.

### Measurement methods

The patients in our study had dinner on the night before the examination and then fasted. They visited the hospital on the morning of the examination under fasting conditions. After visiting the hospital, they could defecate and urinate; they were then rested for 30 minutes. Measurements were performed within 1 hour thereafter. The BMI was calculated as weight/height^2^. InBody720 (Biospace Co. Ltd., Seoul, Korea) was used as the bioelectrical impedance analyzer to measure skeletal muscle mass. The patients were positioned in a standing posture, thereby facilitating close contact of a total of eight electrodes with both the hands and feet. Weak, noninvasive alternating current flows from these electrodes. The impedance of each limb and fuselage was measured, and the appendicular muscle mass of each limb was estimated from this impedance [14, 15].

Based on skeletal muscle mass by site, the appendicular skeletal muscle mass index (SMI) was calculated using appendicular skeletal muscle mass/height^2^. A decrease in muscle mass was defined as an SMI of ≤6.57 kg/m^2^ in men and of ≤4.94 kg/m^2^ in women [16].

Slim Vision Ver3 (Cybernet Systems Co. Ltd., Tokyo, Japan), a software that calculates abdominal computed tomography images obtained at the navel level, was used for measuring the visceral fat area. Patients with a visceral fat area of ≥100 cm^2^ were defined as having visceral obesity [17]. Gait speed was measured using a 10-m gait test. Patients were asked to walk at a normal gait speed for 14 m, and the time required for walking 10 m after walking 2 m from the starting point was measured. Measurements were performed twice, and the mean values of the two measurements were used. The cut-off value of the 10-m gait test was 0.8 m/s [2]. The dominant hand was used to measure grip strength. Measurements were performed twice while patients were in the standing position, and the mean value of the two measurements was used. The cut-off values of grip strength were 26 kg for men and 18 kg for women [2].

The amount of food intake was determined via an interview with a dietitian. During outpatient visits, the patients were comprehensively asked what they ingested in the past 24 h. The energy (kcal) of each food ingested was evaluated using Standard Tables of Food Composition in Japan, 2015 (Seventh Revised version) [18]. Subsequently, the total energy (kcal) of food intake in the past 24 h was evaluated, which is expressed as percentage values when the preoperative levels were adjusted to 100%.

### Diagnostic criteria for sarcopenia, obesity, and visceral obesity

Sarcopenia was diagnosed when the 10-m gait speed or grip strength and the SMI were below the cut-off values [2, 19]. The cut-off value for SMI was ≤ 6.57 kg/m^2^ in men and ≤ 4.94 kg/m^2^ in women [16]. Obesity was diagnosed when BMI was > 25 kg/m^2^ [19], whereas visceral obesity was diagnosed when the abdominal visceral fat area was > 100 cm^2^ [17].

The present study was conducted in accordance with the Declaration of Helsinki and was approved by the Institutional Review Board of National Hospital Organization Hamada Medical Center. The content of the study was explained orally and in writing to all the patients. Written informed consent was obtained from all patients.

### Statistical processing

Measured values are denoted as mean ± standard deviation (mean ± SD). Bartlett’s test was used for the test of variance among the three groups of continuous variables. Two-factor analysis of variance was used to test multiple groups for continuous variables, and Tukey’s method was used for post hoc testing. Further, the Cochran’s Q test was used to test multiple groups for nominal variables, and the Steel–Dwass method was used to test for post hoc testing. Pearson’s correlation coefficient was used to test correlation. Statistical significance was defined as p < 0.05. EZR ver. 1.34 (Saitama Medical Center, Jichi Medical University) was used to perform all statistical analyses [20].

## Results

The subjects were 50 patients [32 men and 18 women; mean age, 77 ± 6.7 years (mean ± standard deviation)]. The mean postoperative course at the time of the 6-month postoperative examination was 6.6 ± 0.82 months and that at time of the 1-year postoperative examination was 1.2 ± 0.95 years. The preoperative weight of the patients was significantly decreased at 6 months postoperatively and significantly increased at 1 year postoperatively. The preoperative BMI significantly decreased at 6 months postoperatively and significantly increased at 1 year postoperatively. No significant differences were noted for grip strength or serum albumin levels between 6 months and 1 year postoperatively. Gait speed significantly decreased at 6 months postoperatively (Table 1).

**Table 1:** Measurement results preoperatively, 6 months postoperatively, and 1 year postoperatively.

		Preoperatively	6 months after gastrectomy	1 year after gastrectomy
**Body weight (kg)** ^†^	**Men**	59.4 ± 8.6^a^^,^^c^	49.8 ± 8.2^a^^,^^b^	53.6 ± 8.0^b^^,^^c^
	**Women**	54.7 ± 7.6^a^^,^^c^	46.1 ± 6.6^a^^,^^b^	49.4 ± 7.2^b^^,^^c^^,^
**Body mass index (kg/m**^**2**^**)** ^†^	**Men**	23.8 ± 3.2^a^	20.2 ± 2.8^a^^,^^b^	22.3 ± 3.0^b^
	**Women**	22.6 ± 3.4^a^	17.4 ± 2.6^a^^,^^b^	21.1 ± 2.7^b^
**Serum albumin(g/dl)**	**Men**	4.2 ± 0.3	4.0 ± 0.4	4.1 ± 0.4
	**Women**	4.4 ± 0.3	4.2 ± 0.3	3.9 ± 0.3
**Gait speed (m/s)** ^†^	**Men**	1.78 ± 0.36^a^^,^^c^	1.52 ± 0.45^a^	1.42 ± 0.41^c^
	**Women**	1.74 ± 0.32^a^^,^^c^	1.46 ± 0.51^a^	1.39 ± 0.48 ^c^
**Grip strength (kg)**	**Men**	27.4 ± 5.1	26.5 ± 5.0	26.6 ± 5.1
	**Women**	19.3 ± 6.2	17.6 ± 4.8	17.7 ± 4.6
**Visceral fat area (cm**^**2**^**)** ^†^	**Men**	128.6 ± 41.2^a^^,^^c^	76.2 ± 41.0^a^^,^^b^	96.2 ± 40.2^b^^,^^c^
	**Women**	122.0 ± 78.5^a^^,^^c^	72.3 ± 35.2^a^^,^^b^	91.4 ± 34.3^b^^,^^c^
**Appendicular skeletal muscle**	**Men**	8.25 ± 1.72^a^^,^^c^	6.92 ± 1.49^a^	7.07 ± 1.55^c^
**mass index (kg/m**^**2**^**)** ^†^	**Women**	6.21 ± 1.67^a^^,^^c^	5.31 ± 1.66^a^	5.54 ± 1.68^c^
**Amount of food intake (%)**^††^	**Men**	100^a^^,^^c^	80 ± 5.2^a^^,^^b^	85 ± 5.9^b^^,^^c^
	**Women**	100^a^^,^^c^	74 ± 6.1^a^^,^^b^	82 ± 6.1^b^^,^^c^

Values are presented as mean ± standard deviation

The amount of food intake is expressed as % when the preoperative levels were adjusted to 100%.

^†^Two-factor analysis of variance revealed significant differences among the three groups (preoperatively, 6 months after gastrectomy, and 1 year after gastrectomy) (p < 0.05).

^††^ Cochran’s Q test revealed significant differences among the three groups (p < 0.05).

^a^significant differences in values between preoperatively and 6 months after gastrectomy (p < 0.05).

^b^significant differences in values between preoperatively and 1 year after gastrectomy (p < 0.05).

^c^significant differences in values between 6 months after gastrectomy and 1 year after gastrectomy (p < 0.05).

The preoperative visceral fat area was significantly decreased at 6 months postoperatively and significantly increased at 1 year postoperatively. The preoperative SMI was significantly decreased at 6 months postoperatively and showed a slightly increasing trend during the 1-year postoperative period. A comparison of the SMI at 6 months and 1 year postoperatively did not reveal any significant difference (Table 1).

Dietary intake is expressed as percentage values when the preoperative levels were adjusted to 100%, which significantly decreased to 78% 6 months postoperatively and significantly increased at 1 year postoperatively (Table 1).

With regard to weight, gait speed, BMI, SMI, and visceral fat area, there were significant differences between men and women at the same measurement time (Table 1).

**Fig 1:**
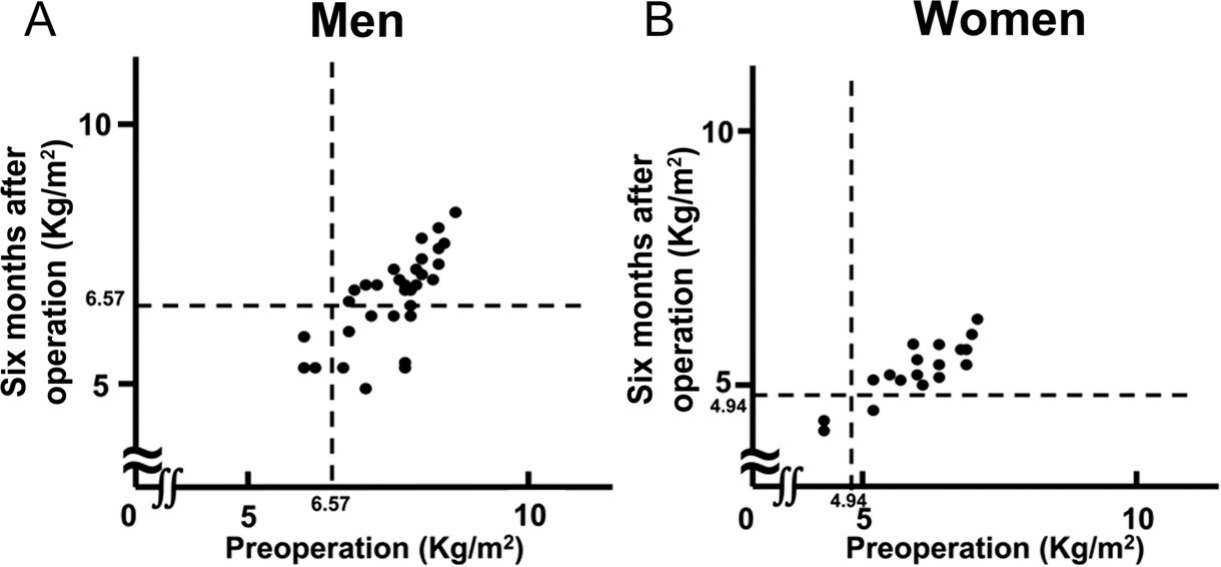
Change in SMI preoperatively and 6 months after gastrectomy. SMI: appendicular skeletal muscle mass index 6.57: SMI cut-off value for diagnosing sarcopenia in men. 4.94: SMI cut-off value for diagnosing sarcopenia in women.

In men, the preoperative SMI was less than the cut-off value in 9.4% of the patients; their preoperative SMI values remained less than the cut-off value at 6 months postoperatively. In 25% of the patients, the SMI was below the cut-off value at 6 months postoperatively (Fig 1).

In women, the preoperative SMI was less than the cut-off value in 11% of the patients. Similar to that in men, the preoperative SMI in these patients was less than the cut-off value at 6 months postoperatively. The SMI dropped immediately below the cut-off value in 5.5% of the patients at 6 months postoperatively (Fig 1).

**Fig 2:**
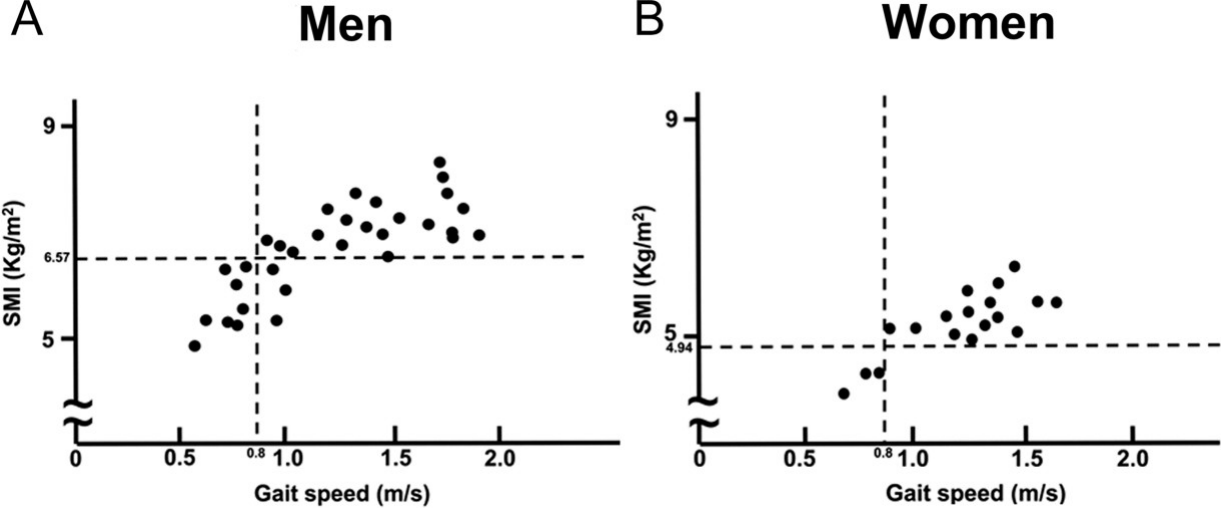
Relationship between gait speed and SMI at 1 year after gastrectomy. SMI: appendicular skeletal muscle mass index 6.57: SMI cut-off value for diagnosing sarcopenia in men. 4.94: SMI cut-off value for diagnosing sarcopenia in women. 0.8: gait speed cut-off value for diagnosing sarcopenia.

In men, the gait speed was ≤ 0.8 m/s in 34.3% of the patients. Among these, SMI was less than the cut-off value in 72.7% of the patients at 1 year postoperatively (Fig 2).

In women, the gait speed was ≤ 0.8 m/s in 16.6% of the patients. The SMI in these patients was below the cut-off value at 1 year postoperatively (Fig 2).

**Fig 3:**
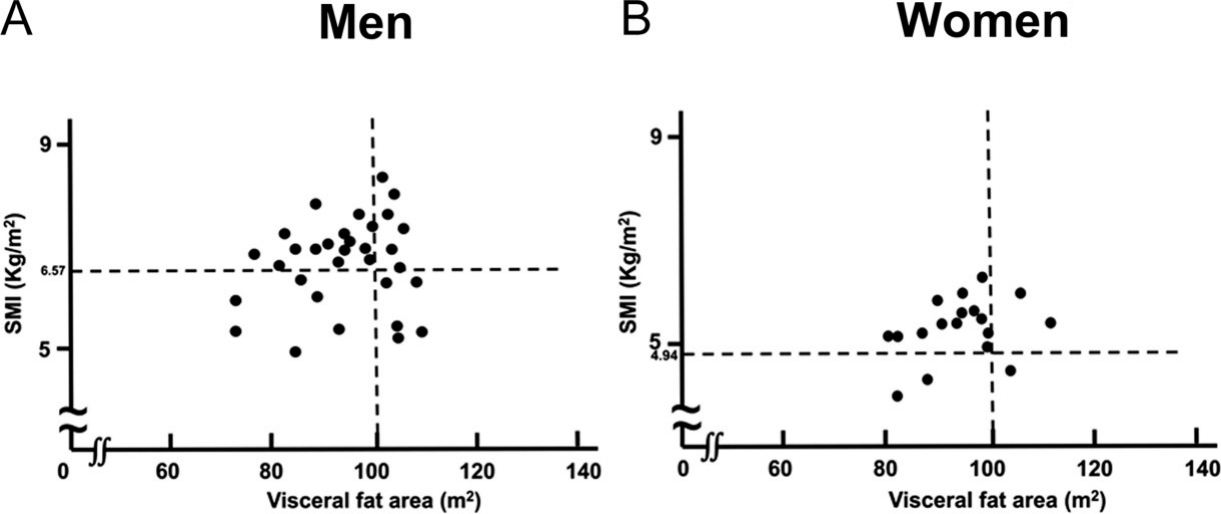
Relationship between the visceral fat area and SMI one year after gastrectomy. SMI: appendicular skeletal muscle mass index 6.57: SMI cut-off value for diagnosing sarcopenia in men. 4.94: SMI cut-off value for diagnosing sarcopenia in women. There was no correlation between the visceral fat area and SMI at 1 year postoperatively in men (r = 0.048, *p* = 0.821) and women (r = 0.376, *p* = 0.150).

An examination of the relationship between visceral fat area and SMI at 1 year postoperatively revealed no correlation in men (r = 0.048, *p* = 0.821) and women (r = 0.376, *p* = 0.150; Fig 3). The visceral fat area at 1 year postoperatively was above the cut-off value in 34.4% men. Among these, 45.5% of the patients had an SMI below the cut-off value (Fig 3). In women, the visceral fat area at 1 year postoperatively above the cut-off value of 100 cm^2^ in 16.7% of the patients. Among these, 33.3% patients had an SMI below the cut-off value (Fig 3).

Preoperatively, 6% of the patients were diagnosed with sarcopenia and this increased to 20% by 6 months postoperatively. Further, 2% of the patients were diagnosed with obesity at 6 months postoperatively and this increased to 8% by 1 year postoperatively; however, this increase was not significant. At 6 months postoperatively, 12% of the patients were diagnosed with visceral obesity that increased to 28% by 1 year postoperatively, and 2% of the patients were diagnosed with sarcopenia and visceral obesity at 6 months postoperatively that increased to 12% by 1 year postoperatively (Table 2).

**Table 2:** Number of patients with sarcopenia, obesity, and visceral obesity.

	Preoperatively	6 months after gastrectomy	1 year after gastrectomy
**Sarcopenia**^†^	6% (3)^a^	20% (10)^a^	22% (11)
**Obesity**^†^	28% (14)^a^	2% (1)^a^	8% (4)
**Visceral obesity**^†^	38% (19)^a^	12% (6)^a^^,^^b^	28% (14)^b^
**Sarcopenia and visceral obesity**^†^	2% (1)	2% (1)^b^	12% (6)^b^

^†^Cochran’s Q test revealed significant differences among the three groups (p<0.05).

^a^significant differences in values between preoperatively and 6 months after gastrectomy (p < 0.05).

^b^significant differences in values between 6 months after gastrectomy and 1 year after gastrectomy (p < 0.05).

## Discussion

Research on sarcopenia has recently progressed, but its definition and diagnostic criteria have not yet been standardized. In the present study, the AWGS diagnostic criteria were used as reference [2]. The walking speed and grip strength were measured as defined in AWGS because the measured values (AWGS) of those Japanese individuals have been published. However, the method to measure muscle mass has not yet been standardized, and there are no standard values for Japanese people. Moreover, the mean and median values of muscle mass vary according to race, sex, age, etc. Therefore, it is difficult to use reference values used in Western and other Asian countries. In the present study, bioelectrical impedance analysis was used to measure muscle mass. When a bioelectrical impedance analyzer is used, even if the muscle mass of the same person is measured, there is a difference depending on the instrument and measurement method used.

Regarding the reference values used for the diagnosis of sarcopenia, the European Working Group on Sarcopenia in Older People recommends obtaining a reference value from a normal group (healthy young adults) rather than other elderly groups and setting the cut-off value as (the mean value − 2 standard deviations) [1]. Therefore, in the present study, the cut-off value for muscle mass used for diagnosing sarcopenia was determined using the mean value and standard deviation of muscle mass for healthy subjects aged 18–40 years who had undergone measurements at Hamada Medical Center [16].

Sarcopenic obesity is a state in which sarcopenia and obesity coexist and has recently been gaining attention in relation to metabolic syndrome [21]. It is a condition in which the risk of developing lifestyle diseases such as diabetes, hyperlipidemia, and hypertension is high as the body function decreases [22]. However, unlike diagnostic sarcopenia, the diagnostic criteria for sarcopenic obesity are not unified [22]. The characteristics of sarcopenic obesity include increasing body fat and decreasing skeletal muscle mass. To diagnose sarcopenic obesity, body fat and skeletal muscle mass must be considered [19]. As shown in Fig 3, there was no correlation between visceral fat area and SMI. Therefore, it is recommended that body fat and skeletal muscle mass should to be separately evaluated.

Although the diagnosis of obesity is often judged based on BMI, BMI does not reveal an increase in body fat or a decrease in skeletal muscle mass [19, 23]. Further, when obesity is determined based on the measured value of BMI, BMI is only considered to have modest validity correlated with cardiovascular risk factors [24]. When visceral fat accumulation is determined from the measured value of waist circumference or waist/hip ration, abdominal adiposity is considered a strong measurement value correlated with the risk factors of cardiovascular and metabolic syndrome [24, 25]. Further, visceral fat accumulation is associated with risk factors for atherosclerosis in nonobese Japanese people [26]. In this study, we investigated the frequency of sarcopenia, obesity, and visceral obesity.

Unlike the method of measuring muscle mass, the method for measuring visceral fat accumulation has not yet been standardized, and there are no standard values for Japanese people. Moreover, visceral fat has different mean and median values depending on race, sex, age, etc. The Guidelines for the Management of Obesity Disease 2016 recommends the use of computed tomography for the measurement of the visceral fat area at the umbilical level [17]. In the present study, patients with an abdominal visceral fat area of ≥100 cm^2^ were defined as having visceral obesity; this is the cut-off value of visceral fat accumulation for diagnosing metabolic syndrome in Japan [17].

After distal gastrectomy, stomach volume as well as food consumption decreases, thereby resulting in weight loss during the perioperative period [10], which was also observed in our present study. In a state in which oral ingestion does not progress, energy intake is insufficient and an increase in muscle mass cannot be expected. Studies have examined the effectiveness of enteral nutrition as a countermeasure [27–30]; however, studies still report perioperative weight loss and muscle loss. To the best of our knowledge, no study has reported the effect of increasing only the protein intake to stop the decrease in muscle mass after gastrectomy. The increased protein intake along with the limited oral intake ensures that the absolute and relative intake levels of lipids and carbohydrates are reduced. The influence on living organisms in such a state is largely unknown.

In the present study, compared with preoperative values, the SMI decreased 6 months postoperatively. If the muscle mass decrease until 6 months postoperatively can be inhibited, secondary sarcopenia can be prevented. In recent years, the concept of enhanced recovery after surgery has spread to patients undergoing gastrectomy, and nutritional therapy and rehabilitation from the early postoperative period are recommended [17]. No differences regarding postoperative complications have been noted in comparison with conventional postoperative management, but effects such as reduction in hospitalization duration and medical costs have been noted [31, 32]. At our hospital, we are actively engaged in the concept of enhanced recovery after surgery, but the decrease in muscle mass during the perioperative period could not be stopped in our patients.

During the perioperative period, as a result of stress associated with treatment, protein catabolism is promoted and protein assimilation is suppressed. This is a defense reaction of the body against stress, which remains difficult to control [33]. Furthermore, the ratio of endogenous and exogenous energy expended during the study period is unknown. For this reason, exogenous energy cannot be effectively used even if the body is extensively replenished with only exogenous energy [34]. Thus, it is necessary to accept some reductions in weight loss and muscle mass during the perioperative period.

In the present study, the frequency of sarcopenia did not change 1 year postoperatively compared with 6 months postoperatively, but an increasing visceral obesity was noted. At 6 months postoperatively, food intake was increased and the decrease in body weight was halted. A comparison of the findings at 6 months and 1 year postoperatively revealed a significant increase in visceral fat mass, but no significant increase in SMI was noted. In other words, the increase in weight over the period from 6 months to 1 year postoperatively is mostly accounted in terms of fat. Therefore, the state of having sarcopenia and visceral obesity was found to be increasing at 1 year postoperatively. These values cannot be judged based on BMI and amount of food intake, and it is necessary to measure skeletal muscle and abdominal visceral fat.

It has been reported that resistance training is effective for sarcopenia in elderly people [35, 36]. Furthermore, the necessity of complex intervention with nutritional intervention, including amino acid supplements, has been indicated [37]. Such complex intervention may be effective for preventing sarcopenia in elderly people following gastrectomy. However, there are many unclear points concerning the safety and efficacy of complex intervention in patients who have undergone gastrectomy as food intake is less in such patients. Nevertheless, if the food intake is improved and weight gain is noted, complex intervention may be effective for preventing sarcopenia.

## Conclusions

To date, the main purpose of follow-up after gastrectomy for gastric cancer has been surveillance for early detection of recurrence and secondary cancer. However, in recent years, attention has also been paid to cope with postgastrectomy symptoms and overcome nutritional issues [13]. Few studies have reported the presence of sarcopenia and its long-term characteristics following gastrectomy for gastric cancer. We believe that henceforth, the importance of evaluating postoperative sarcopenia and visceral obesity must be considered. In particular, sarcopenia and visceral obesity after increases in BMI and food consumption should be carefully monitored.
